# An Integrated Multi-Functional Thermal Conductive and Flame Retardant Epoxy Composite with Functionalized Carbon Nitride Nanosheets

**DOI:** 10.3390/polym15143143

**Published:** 2023-07-24

**Authors:** Yuxin Yang, Ruiping Wang, Yang Leng, Jingchun Wang, Miaojun Xu

**Affiliations:** 1 Aulin College, Northeast Forestry University, Harbin 150040, China; yangyuxin1012@163.com; 2 Heilongjiang Key Laboratory of Molecular Design and Preparation of Flame Retarded Materials, College of Chemistry, Chemical Engineering and Resource Utilization, Northeast Forestry University, Harbin 150040, China; wangrp0916@163.com (R.W.); wangjingchun0218@163.com (J.W.)

**Keywords:** carbon nitride nanosheets, piperazine pyrophosphate, thermal conductivity composites, flame retardancy

## Abstract

In miniaturized and integrated electronic devices, thermal potential and fire hazards caused by heat diffusion require an efficient thermal management system with versatile electronic packaging equipment. The flame retardancy was endowed on the surface of carbon nitride after thermal etching (CNNS) containing piperazine pyrophosphate (PPAP) by hydrogen bonding, and the obtained nanosheet was defined as PPAP-CNNS. During solution blending and program-controlled curing, PPAP-CNNS was used as a multifunctional filler to fabricate highly thermoconductive and fire retardant epoxy resin (EP) composites. In line with expectations, the resultant EP composites containing 7 wt% PPAP-CNNS had an exceptional thermal conductivity (TC) of 1.1 W·m^−1^K^−1^, which was 4.8 times higher than pure EP. Simultaneously, there was a sharp drop in the heat release rate (HRR), total heat release (THR), smoke production rate (SPR), and total smoke production (TSP) compared to pure EP. These reductions were, respectively, 63.7%, 54.2%, 17.9%, and 57.2%. The addition of PPAP-CNNS increased the specific surface area, which increased the heat conduction routes, and also the shape of the compact and solid char layer during burning, protecting the underlying polymer. These improvements to dispersion and surface functionalization were made possible by the compound. These results indicate that the preparation of integrated multi-functional resin described in this study has a wide application.

## 1. Introduction

The current miniature integrated electronic equipment generate fierce heat per unit area, which cause device failure and an increased fire hazard in working life [1,2]. Thus, generation of composites that are used as electronic packaging materials simultaneously with a high TC and excellent flame retardant properties is key to guaranteeing the working lifetime and safety. EP is widely used as an electronic encapsulation material due to its ultra-light mass, low cost, and flow processing [3,4,5,6]. However, the evident low thermal conductivity (TC < 0.2 W·m^−1^K^−1^) and inherent flammability limit the heat dissipation materials application in advanced electronic products [7,8]. Consequently, it is extremely imperative and actually valuable to develop materials with a simultaneously high TC and excellent flame retardant properties.

In recent years, research scholars have developed high TC EP materials by incorporating inorganic fillers into the matrix [9,10,11]. Carbon-based nanomaterials, such as graphene and carbon nanotubes (CNTs), were examined to be outstanding thermal conducting fillers due to their elevated TC and being lightweight [12,13,14]. However, the electrical conductivity and expensive price of carbon-based nanomaterials limited their application in electronic equipment. Graphite carbon nitride (g-C_3_N_4_) is a type of two-dimensional polymer material, which, similar to graphene, has aroused extensive research attention due to its high specific surface area, excellent thermal conductivity (>200 W·m^−1^K^−1^), and superior thermal stability [15,16]. The structure of g-C_3_N_4_ has a strong covalent bond and a short bond length, which is similar to h-BN, g-C_3_N_4_, making it a promising electrical insulating material for the thermal management of electronic devices. Dong et al. [16] explored the thermal properties of g-C_3_N_4_ through non-equilibrium molecular dynamics simulations, and the thermal conductivity of g-C_3_N_4_ reached 49.8 and 111.9 W·m^−1^K^−1^, respectively. The g-C_3_N_4_ is introduced as a thermally conductive filler into EP, which passes through the NH_2_ and NH groups in g-C_3_N_4_. Wu et al. [17] prepared a g-C_3_N_4_/nanofiber cellulose thermal conductive film filler to improve the thermal conductivity of polydimethylsiloxane, and its thermal conductivity was 1.94 W·m^−1^K^−1^. However, the versatility of the above composites was ignored.

To improve the flame retardant performance of EP, silicon [18], nitrogen [19], phosphorus–nitrogen [20], metal hydroxides [21], and transition metal oxides [22] have been demonstrated to be effective synergistic flame retardants. Phosphorus–nitrogen compounds stood out among them for their effectiveness as flame retardants, their low toxicity, and environmental friendliness [23,24,25]. According to former reported works, polyphosphoric acid piperazine (PPAP) [26,27,28] was found to have a high flame retardant efficiency for EP thermosets. Hu et al. [26] applied the preparation of flame retardant polyamide 6 by mixing PPAP with aluminum hypophosphite (AHP) and the samples fulfilled the UL-94 V-0 rating when PPAP/AHP was added in an 18 weight percent ratio. The results showed that PPAP had a strong flame retardant impact on polyamide composites while they were in the condensed phase. On the other hand, it has been demonstrated that the combined effects of the thermal conductivity filler and flame retardant filler can increase the fire resistance of polymers without destroying their mechanical and thermal properties [29]. Their thermal conductivity filler and synergistic flame retardation mechanisms, which include free radical adsorption [30], physical barriers [31], and the catalytic carbonization effect [32], etc., were considered to contribute the enhancing flame retardancy. Feng et al. [33] revealed a synergistic increment in the TC of epoxy/hexagonal born nitride (hBN)@graphene oxide (RGO)@Ni(OH)_2_ composites (39.4% increment to 2.01 W·m^−1^K^−1^) by introducing 20 wt% hBN with 2 wt% RGO@Ni(OH)_2_. With a high LOI value of 29.2% and a V-1 burning rate, the outcome demonstrated good fire resistance. Nonetheless, the high addition amount of these mixed fillers and the weak compatibility between the mixed filler with the thermal conductive and flame retardant properties and EP seriously damaged the mechanical performance of EP composites and extremely limited their development in the booming application fields [34,35,36]. Therefore, the secret to creating high-performance composites is determine how to combine a filler with outstanding thermal conductivity and flame retardant qualities with addition in low amounts.

Thus, using a straightforward thermal oxygen etching process, carbon nitride nanosheets (CNNS) with extremely large individual surface areas and good dispersion were created, which were subsequently combined with PPAP. High-performance EP/PPAP-CNNS thermosets were prepared using synergistic fillers with diethylenetriamine (DETA) as the curing agent to simultaneously improve TC and flame retardancy through multiple synergistic effects. In this work, a novel and promising hybrid casting technique to create functional epoxy resin has been developed.

## 2. Materials and Methods

### 2.1. Materials

**Scheme 1:**
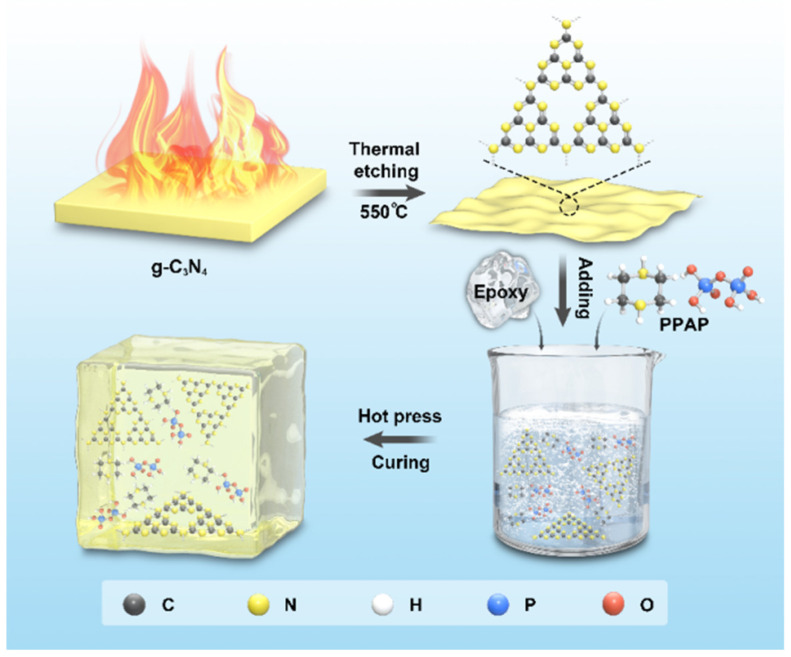
Schematic diagram of the preparation process of carbon nitride nanosheets.

Diglycidyl ether of bisphenol-A epoxy (EP, E-51, epoxy equivalent: 184~194 g·eq^−1^, viscosity: 11,000~14,000 m·Pa·s) was supplied by Xingchen Synthetic Material Co., Ltd., (Nantong, China). Diethylenetriamine (DETA), used as a cured agent, was purchased from Aladdin Industrial Corporation (Shanghai, China). Piperazine pyrophosphate (PPAP) and g-C_3_N_4_ were made in the laboratory according to our previous work [18]; the chemical structure of PPAP is illustrated in Scheme 1.

### 2.2. Preparation of Carbon Nitride Nanosheets

The carbon nitride nanosheets were synthesized with g-C_3_N_4_ as the raw material by direct thermal exfoliation at 550 °C for 4 h under an air atmosphere (a weak oxidizing atmosphere); the resultant products were denoted as CNNS. The obtained sample was pale yellow.

### 2.3. Preparation of CNNS/Epoxy Composites, PPAP/Epoxy Composites, and CNNS/PPAP/Epoxy Composites

A variety of CNNS/epoxy composites were created using casting and molding methods. Initially, epoxy resin and CNNS were mechanically stirred in a beaker at 25 °C for 20 min. After that, DETA, which served as a curing agent, was vigorously stirred into the epoxy resin and CNNS combination. Additionally, a weight ratio of around 100/8 was maintained between the epoxy resin and curing. The EP resin, CNNS, and DETA mixture was then well mixed at 25 °C for 10 min. The CNNS/EP mixture was finally poured into the mold and subjected to a programmed curing at 50 °C for 1 h and 80 °C for 2 h. Similarly, EP composites with other filler ratios were prepared using the same procedure, denoted by EP (without filler), PPAP/EP, and CNNS/PPAP/EP (CNNS and PPAP were thoroughly ground and mixed before casting).

### 2.4. Characterization

The samples were tested using a Fourier infrared spectrometer model PerkinElmer 400 (Waltham, MA, USA). The test samples were solids, and the infrared potassium bromide compression method was used by taking approximately 10 mg of the sample and mixing it with potassium bromide solid, grinding it thoroughly, placing it into a sample-forming mold, and obtaining the test samples at a pressure of 20 MPa for 1 min, setting a test wavelength range of 4000–400 cm^−1^ and repeating the scanning three times.

The specific surface area of the CNNS particles was tested by the N2 adsorption/desorption measurements using a Belsorp-mini (BEL, Osaka, Japan) at 77 K.

Using the CZF-3 equipment (Vouch, Jiangning, China) and a 130 mm × 13 mm × 3.2 mm sized sample, UL-94 tests were performed in compliance with the specification. The JF-3 Oxygen Index Instrument (Nanjing Tiance Technology Co., Ltd, Nanjing, China) was used to measure the limiting oxygen index (LOI) data in accordance with the GB/T 2406-93 standard using a sample sized 130 mm × 6.5 mm × 3 mm.

A conical calorimeter generated by the Fire Testing Technology CONE (Suzhou, China) was used for the conical calorimetric test. The sample size was 100 mm × 100 mm × 4.0 mm, and it was put to the test using a heat flow of 50 kW·m^−2^ in accordance with the ISO-5660 standard.

A FEIQUANTA-200 SEM (Thermo Fisher Scientific, Eindhoven, The Netherlands) was used to examine the EP thermosets and leftovers following CONE testing using scanning electron microscopy (SEM). Surface scanning model underwent an elemental analysis by EDX. Before the charcoal layer morphology was evaluated, all of the samples were glued to a copper plate for gold spraying, and the accelerating voltage was set to 20 kV to view the sample morphology at various magnifications.

A PerkinElmer Pyris 1 Thermal Analyzer (PerkinElmer, Boston, MA, USA) was used to conduct thermogravimetric analyzer (TGA) tests. Real-time recordings of the sample cracking gas infrared were taken as the temperature was ramped up over the temperature range of 50–800 °C, at a flow rate of 20 mL·min^−1^ under a nitrogen environment. The sample mass was roughly 3–8 mg.

The DMA8000 thermal analysis system (PerkinElmer, Boston, MA, USA) was used to conduct dynamic mechanical analysis (DMA) testing. The sample size was a 40 mm × 10 mm × 4 mm specimen heated from 30 °C to 200 °C at a rate of 3 °C·min^−1^ in an air environment. The sample was evaluated using a double cantilever device at 1 Hz, 4 N, and 2 m.

X-ray photoelectron spectroscopy analysis (XPS) data were performed using a thermo spectrometer (Waltham, MA, USA) consisting of a Kα hemispherical electron analyzer and a monochromated AlKα source. Tests were carried out in an ultra-vacuum regime for the elements C, N, O, and P.

A model confocal DXR2 Raman spectroscopy equipment (Bruker, Karlsruhe, Germany) was used to conduct the Laser Raman Spectroscopy (LRS). A 532 nm Helium-Neon laser (Alphalas, University of Göttingen City, Germany) was used for the experiments, which were carried out at room temperature. Samples were taken from the leftover charcoal from the CONE tests.

C-Therm Technologies (Vancouver, BC, Canada) measured the thermal conductivity of composites at room temperature. The samples were cylinders of 30 mm × 30 mm × 5 mm.

A handheld infrared camera (Testo AG, Black Forest, Germany) was used to view the evolution of the temperature distribution of the substrate while it was being heated from 25 to 100 °C on a homemade programmable point heating unit. The EP, CNNS/EP, and CNNS/PPAP/EP splines were placed on a 100 °C platform, and the temperature was recorded every 30 s and photos were taken every minute.

## 3. Results

### 3.1. Characterizations of CNNS

In the past, it was widely accepted that g-C_3_N_4_ had a layered structure akin to that of graphite, with each layer having powerful covalent C-N bonds rather than C-C bonds and weak van der Waals interactions between layers. In this work, CNNS was obtained via a straightforward procedure that involved “etching” g-C_3_N_4_ with direct heat oxidation in air. This approach was based on the fact that the hydrogen bond cohesive chains (especially short ones) in the multilayer polymer melon units were not stable (or reactive) during air oxidation, reducing the thickness of the g-C_3_N_4_ blocked to the required nanoscale [34]. The structural schematic diagram of g-C_3_N_4_ and CNNS are shown in Scheme 1. The CNNS obtained through stripping was light yellow, and despite having the same weight, the volume was substantially more than that of g-C_3_N_4_.

**Figure 1:**
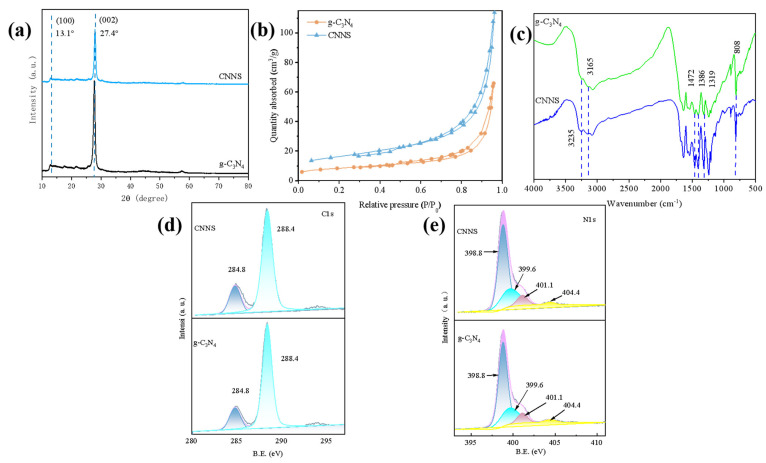
(**a**) XRD patterns, (**b**) BET spectra, and (**c**) FTIR spectra of g-C_3_N_4_ and CNNS. (**d**) C 1s and (**e**) N 1s XPS spectra of g-C_3_N_4_ and CNNS.

The crystal structure of CNNS and g-C_3_N_4_ were further confirmed by X-ray technique. As shown in Figure 1a, the g-C_3_N_4_ has only two obvious diffraction peaks at 2θ = 13.1° and 27.4°, respectively. The strongest 2θ = 27.4° diffraction peak represented the (002) crystal plane of g-C_3_N_4_ and the two-dimensional structure of the conjugated aromatic ring [35]. This crystal plane structure was the structural characteristics of the graphite phase, and g-C_3_N_4_ was also called the graphite-like phase carbon nitride for this reason. The 2θ = 13.1° corresponds to (100) crystal plane diffraction of g-C_3_N_4_, which was caused by interlamellar stacking of azine ring elements. After thermal etching, the (002) peak revealed a remarkably reduced intensity and was slightly offset. However, the thickness remained the same compared to g-C_3_N_4_, suggesting significantly less ordered stacking in the z direction caused by the reduced thickness following thermal etching, which indirectly demonstrates the effectiveness of CNNS exfoliation [36,37]. The crystal structure was not damaged after the thermal etching. However, the diffraction peak at 2θ = 13.1° became less obvious, which was mainly caused by the destruction of the stacking structure of the aromatic ring and the reduction in its size during the stripping process [38].

The Brunauer–Emmett–Teller (BET) specific surface area of the generated samples was determined under nitrogen adsorption–desorption conditions. Figure 1b depicts CNNS with conventional IV isotherms and h3-type hysteresis rings, both of which point to the presence of slit mesopores brought on by nanolayer aggregation. In comparison to g-C_3_N_4_ (17.953 m^2^·g^−1^), CNNS had a BET surface area of 37.941 m^2^·g^−1^. It was further demonstrated that thermal oxidation etching was used to create carbon nitride nanosheets.

Figure 1c displays the findings from an analysis of the structures of g-C_3_N_4_ and CNNS using FTIR. The heptazine ring system was what caused the strong peak at about 810 cm^−1^. N-H stretching was responsible for the large peaks between 3000 and 3600 cm^−1^. Sharper peaks were seen in the range between 900 and 1800 cm^−1^, which were most likely caused by trigonal C-N(-C)-C (complete condensation) or bridging C-NH-C units. These bands demonstrated that the longer strands of hydrogen bond cohered polymeric melon units had survived in a more organized packing following heat oxidation etching in the layers of nanosheets. In addition, the narrowing of the peak reveals that more azimuthal annular units were exposed after stripping, indicating that g-C_3_N_4_ was stripped into CNNS.

XPS was used to analyze the chemical make-up and states of the CNNS and g-C_3_N_4_. According to Figure 1d,e, the core electron binding energies of C and N 1s did not appreciably change, proving that the nanosheet’s carbon and nitrogen chemical states were identical to those of g-C_3_N_4_. As can be seen from Figure 1d, two peaks, C1 and C2, can be fitted for C 1s of g-C_3_N_4_ and CNNS samples at 284.6 and 287.5 eV, respectively, which were derived from the carbon contamination and sp2 hybrid triazine rings in the C-N-C structure, respectively. Figure 1e shows the XPS spectra of N 1s in g-C_3_N_4_ and CNNS. The N 1s in g-C_3_N_4_ and CNNS samples can fit four peaks (N1~N4), which were derived from C-N=C, N-(C)_3_, N-H, and the delocalized π electron and lone pair charge of N 1s in the CN heterocyclic ring, respectively. Based on the percentage of carbon and nitrogen determined by XPS, it was found that the surface atom ratio of carbon to nitrogen increased from 0.88 in the original g-C_3_N_4_ to 0.94 in the nanosheet. Since the nitrogen concentration in the nanosheet surface was obviously low, it may be concluded that the thermal oxidation etching process preferentially oxidizes nitrogen before removing carbon.

### 3.2. Thermal Conductivity of EP Composites

**Figure 2:**
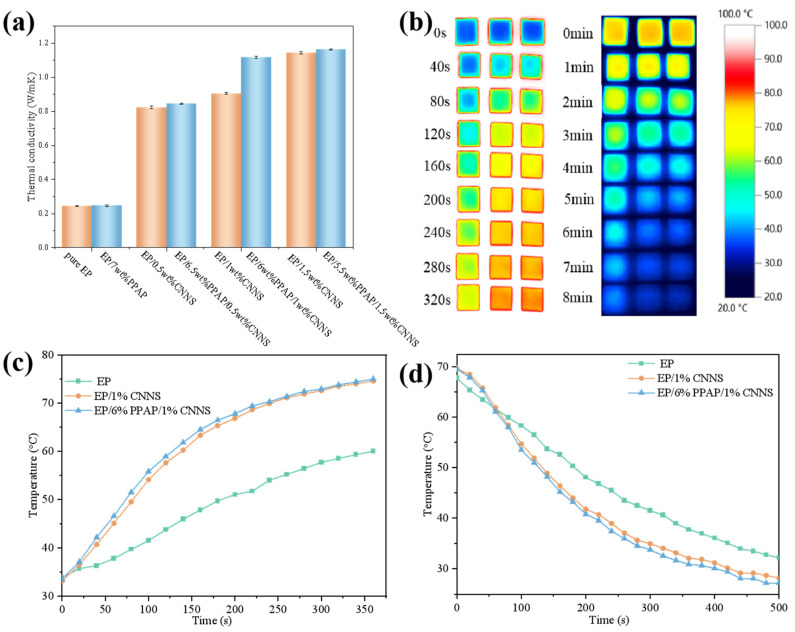
(**a**) Thermal conductivity, (**b**) infrared thermal images, and (**c**) surface temperature variation with heating time and (**d**) cooling time of pure EP, EP/1 wt%CNNS, and EP/6 wt%PPAP/1 wt%CNNS composites.

Figure 2a shown the thermal conductivity curves of EP/g-C_3_N_4_ and EP/CNNS composites with different amounts of thermal conductivity filler and pure EP. One can see that the thermal conductivity of pure EP was only 0.245 W·m^−1^K^−1^ because of its amorphous nature. With the increasing amount of g-C_3_N_4_ and CNNS, the thermal conductivity of EP/g-C_3_N_4_ and EP/CNNS composites increased continuously, showing a positive proportional relationship on the whole. The modification impact of CNNS, with the same amount of CNNS, on the thermal conductivity of composites in the entire experimental system was obviously superior to that of g-C_3_N_4_ without modification. When the addition amount was 1.5 wt%, the thermal conductivity of EP/CNNS was 1.144 W·m^−1^K^−1^, which was 367% higher than that of pure EP. However, the thermal conductivity of EP/g-C_3_N_4_ system was only 0.299 W·m^−1^K^−1^ under the same addition amount, which was only 22% higher than pure epoxy resin. These results mean that the lamellar g-C_3_N_4_ in epoxy resin was gathered together in large quantities due to severe agglomerations and weak interfaces, which formed an effective conduction pathway for phonons and high interfacial resistance [36]. Comparatively, EP/CNNS composites were significantly more effective at increasing TC than g-C_3_N_4_ at the same level of addition. This was due to the effective dispersion and robust interfacial interaction of CNNS in the epoxy resin matrix, which enabled the overlap of the nanosheet fillers and the development of a networked thermal conduction channel.

An infrared thermal imager was used to capture changes in the surface temperature over time during heating and cooling in order to show how EP composites actually behave when it comes to thermal management. The results are displayed in Figure 2b–d. During the heating process, the square samples of pure EP, EP/1 wt%CNNS, and EP/6 wt%PPAP/1 wt%CNNS were placed on a hot table at 80 °C from left to right, respectively. From Figure 2b,c, it can be seen that the surface temperature of all samples continuously increases within the 320 s time and gradually achieves a saturated temperature. Among them, the EP/PPAP/g-C_3_N_4_ composite displayed the fastest temperature increase and highest temperature value, which is consistent with its thermal conductivities shown in Figure 2a. After heating for 320 s, the surface temperature of the EP/6 wt%PPAP/1 wt%CNNS composite was approximately 74.8 °C, which is 16.7 °C and 0.5 °C higher than pure EP and the EP/1 wt%CNNS composite (Figure 2b). Similarly, to evaluate the heat dissipation performance in the composites, all samples were placed in an 80 °C oven for 30 min to ensure a uniform sample temperature, and then were transferred to a plate to cool at room temperature. One can see that the surface temperature of the EP/PPAP/g-C_3_N_4_ composite decreased with the highest rate, which dropped to 37.4 °C at 480 s (Figure 2d). This evidence indicates that the EP/6 wt%PPAP/1 wt%CNNS composite has an excellent thermal transfer ability.

An infrared thermal imager was used to record the image and temperature every 40 s. One can see that the surface temperature of all samples continued to rise due to thermal radiation from the heating station, and the heating rates of EP/1 wt%CNNS and EP/6 wt%PPAP/1 wt%CNNS were significantly faster than those of pure EP, which was consistent with their thermal conductivity. The heating rate of EP/6 wt%PPAP/1 wt%CNNS was slightly faster than EP/1 wt%CNNS. Similarly, all samples were transported simultaneously to the experimental table where images and temperatures were captured with a thermal imager in order to examine the heat dissipation process. As shown in Figure 2c,d, both the EP/1 wt% CNNS and EP/6 wt%PPAP/1 wt%CNNS composites cooled more rapidly during heat dissipation compared to pure EP, and the rate of heat dissipation depended on their TC properties. The outcomes showed that the EP/6 wt%PPAP/1 wt%CNNS composite performed exceptionally well in terms of heat dissipation.

### 3.3. Thermal Stability and Flame Retardancy of EP/PPAP/CNNS Composites

**Figure 3:**
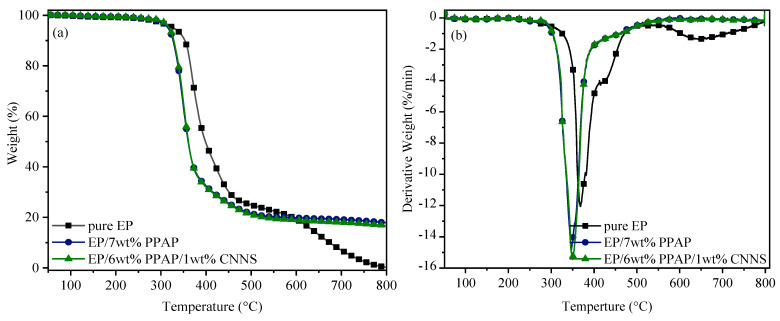
(**a**) TG curves of EP composites under a nitrogen atmosphere. (**b**) DTG curves of EP composites under a nitrogen atmosphere.

One of the most popular methods for quickly assessing the thermal stability of composites is TGA. Figure 3 shows the TGA and DTG curves of pure EP, EP/7 wt%PPAP, and EP/6 wt%PPAP/1 wt%CNNS composites. Table 1 has the related data on thermal deterioration.

Pure EP presented two decomposition peaks at 367.9 °C and 644.6 °C, respectively. Pure EP decomposed at 327.9 °C, and the decomposition rate reached the maximum at 367.9 °C under a nitrogen atmosphere. When the temperature reached 655.6 °C, EP decomposed again, which was due to the low thermal stability of the carbon layer and further decomposition [30,34]. At 800 °C, the carbon residue was 0.3%. It was shown that EP composites have dramatically increased thermal stability. For instance, the TGA curves of EP/7 wt%PPAP showed single-stage degradation with initial and maximum decomposition temperatures of 315.7 and 347.0 °C, respectively, which were reduced by 12 and 20 °C compared to those of pure EP. In addition, when loading for 6 wt%PPAP/1 wt%CNNS, the thermal stability of the EP/PPAP/CNNS composite was similar to EP/7 wt%PPAP. It was noteworthy that EP/7 wt% PPAP and EP/6 wt%PPAP/1 wt%CNNS composites did not continue to decompose, and the carbon residue was 17.9% and 16.7% at 800 °C, indicating the formation of a stable carbon layer. On the one hand, the catalytic carbonation of PPAP and CNNS yielded more carbon residues. On the other hand, the well-dispersed CNNS sheets acts as physical barriers to further inhibit heat transfer.

The composition and related properties of epoxy resin composites were shown in Table 2. It can be seen from Table 1 that when PPAP was added alone, 7% of PPAP can ensure that the epoxy resin material passes UL-94 V-0 in the vertical combustion test, with a limiting oxygen index of 29.7%. When 6% PPAP and 1% CNNS are added, the material can still pass UL-94 V-0 and the limiting oxygen index is 28.8%, indicating that CNNS not only has excellent thermal conductivity, but also exerts a synergistic flame retardant effect.

**Figure 4:**
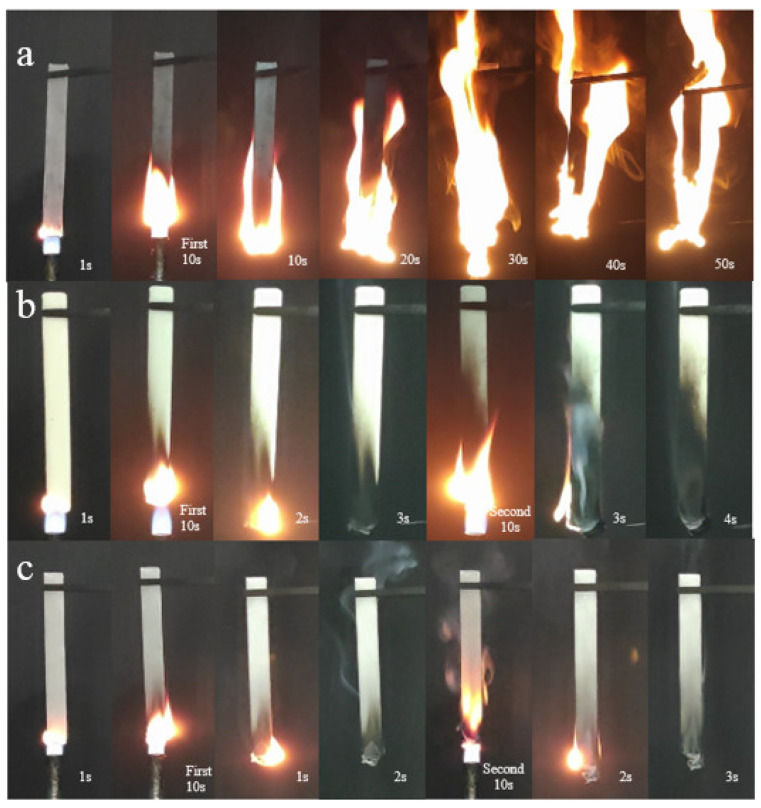
Vertical combustion test screenshots of pure EP (**a**), EP/7%PPAP (**b**), and EP/6%PPAP/1%CNNS (**c**).

Figure 4 displays the outcomes of the vertical combustion test. After the first 10 s of fire, pure EP burns continuously. In contrast to pure EP, specimens that had PPAP or PPAP/CNNS added burned with a smaller flame, demonstrating weaker flame conditions and shorter burning times. The EP/6%PPAP/1%CNNS sample had the weakest flame, and it extinguished itself in the quickest amount of time after being removed from the flame source. The reason was that the CNNS migrated to the surface of the carbon layer during combustion. An effective physical barrier layer was formed inside the matrix to achieve the functions of heat insulation, oxygen isolation, and inhibition of the escape of pyrolysis gas, so as to reduce the release rate of heat, the combustion of materials, and to achieve flame retardancy. According to the aforementioned findings, there is little combustion risk for the EP/PPAP/CNNS composites in a real fire.

**Figure 5:**
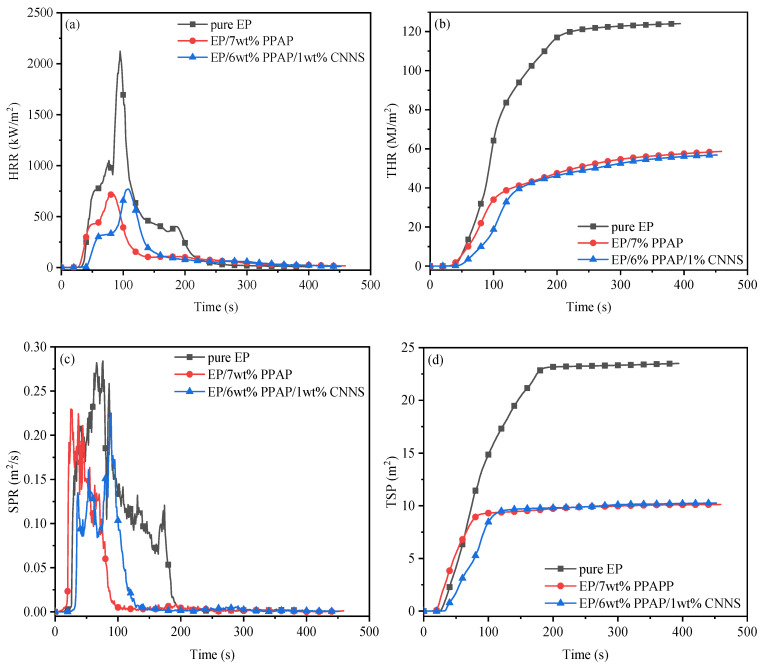
HRR (**a**), THR (**b**), SPR (**c**), and TSP (**d**) curves of pure EP, EP/7%PPAP, and EP/6%PPAP/1%CNNS composites obtained from CONE calorimeter tests at 50 kW·m^−2^.

A CONE calorimeter was used to measure combustion performance in order to more thoroughly characterize the combustion behavior of EP and its composites. The heat release rate (HRR), total heat release (THR), total smoke production (TSP), and smoke production rate (SPR) relationship curves are shown in Figure 5, and the associated combustible characteristics are reported in Table 3.

With a low time to ignition (TTI) of 43 s, a high peak heat release rate (PHRR) of 2123 kW·m^2^, a THR of 118.1 MJ·m^−2^, a high SPR of 0.28 m^2^·s^−1^, and a TSP of 23.5 m^2^, pure EP burned quickly under continuous heat exposure in the CONE test. Incorporating 7%PPAP into EP could effectively slightly decrease the TTI to 29 s, the PHRR value to 723 kW·m^2^, THR to 58.7 MJ·m^−2^, and TSP to 10.1 m^2^, corresponding to a reduction of 65.9%, 53.8%, and 57%, respectively, compared to pure EP. This behavior may be caused by the presence of several acid hydroxyl groups in the PPAP structure, which causes the PPAP to degrade both before and during the initial heating step in the process. The resistance of epoxy thermosets to ignite is interpreted to be weakened by the catalytic breakdown effect of PPAP. Pre-degradation behavior has the advantage of better flame retardation in EP composites under some circumstances by allowing for earlier charging during combustion. More remarkably, when the PPAP/CNNS was removed, the PHRR, THR, SPR, and TSP in the composite showed similar tendencies to those in the EP/PPAP, with corresponding decreases that may reach 63.7%, 51.9%, 21.4%, and 56.2%, respectively, compared to pure EP. The improvement in flame retardancy may be traced to the endothermic effect of CNNS, the dilution effect of PPAP, and the synergistic catalytic carbonization effect of CNNS and PPAP during combustion. Additionally, the PHRR values of EP/7%PPAP and EP/PPAP/CNNS decreased to 723 kW·m^2^ with a reduction of 57.5% at 81 s (tp) and 771 kW·m^2^ with a reduction of 54.7% at 108 s (tp), respectively. The tp of EP/6%PPAP/1%CNNS was 27 s longer than that of EP/7%PPAP. The findings show that it is possible to successfully restrict the release of gas products and reduce the fire risk associated with EP composites during initial material combustion by adding CNNS into the layered structure. EP/PPAP/CNNS composites exhibit considerable potential in heat management applications with fire concerns when combined with strong thermal conductivity.

**Figure 6:**
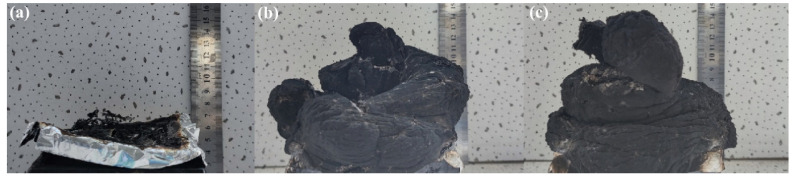
Digital photos of pure EP (**a**), EP/7% PPAP (**b**), and EP/6% PPAP/1% CNNS (**c**) after the CONE test.

The char residues of pure EP, EP/7%PPAP, and EP/6%PPAP/1%CNNS after the CONE calorimeter test were measured by a digital camera and SEM magnified by 2000 times (Figure 5 and Figure 6), in order to further investigate the relationship between the morphology of char layers and flame retardant properties of the EP composites. It is obvious that pure EP cannot function as a barrier or protective layer after combustion because there are fragmented char residues left behind. However, the EP/7%PPAP and EP/6%PPAP/1%CNNS sample showed a continuous, expanded, dense, and complete char layer due to the addition of PPAP, as shown in Figure 5b,c. In order to improve the flame retardant performance of EP composites, the extended char layer was conductive to functioning as an isolation layer, decreasing the exchange of heat and oxygen.

**Figure 7:**
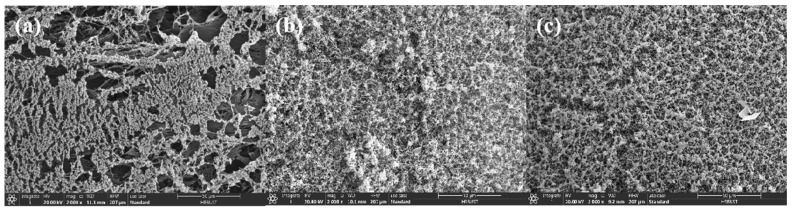
SEM images of the char residue after CONEcalorimeter tests: (**a**) pure EP, (**b**) EP/7%PPAP, and (**c**) EP/6%PPAP/1%CNNS.

Furthermore, Figure 7a shows that the surface morphology of the pure EP char layer exhibits a somewhat flexible structure. This resulted in some surface cracks and holes since there was not enough char layer created during combustion. As a result, pure EP has poor flame retardancy because heat and flammable volatiles could pass through the char layer and into the flame area. On the contrary, when the flame retardant PPAP containing the piperazine structure was introduced, the EP composites burn to produce a more dense, complete, and expanded carbon layer than pure EP, as shown in Figure 7b,c. The findings indicated that the addition of PPAP not only promoted the production of a carbon layer in the EP matrix, but also that the decomposition of PPAP during combustion promoted the formation of an intrinsic char layer, increasing the thickness and density of the char layer. By effectively blocking heat, oxygen, and flammable volatiles from entering the interior of the matrix and from entering the flame zone, the created char layer further delays the deterioration and combustion of the EP matrix. On the other hand, PPAP decomposes to produce phosphorus-containing free radicals, which have a quenching effect and reduce gas phase combustion, thereby obtaining excellent flame retardant properties. In contrast, the chars of EP/PPAP/CNNS composites also revealed the same porous morphologies but with less addition of PPAP than that of the EP/PPAP composite. In the SEM images, the surface char skeletons are shown as compact, integrated, uniformly dispersed char residues with a strong CNNS interface, which might considerably increase the char layer strength.

**Figure 8:**
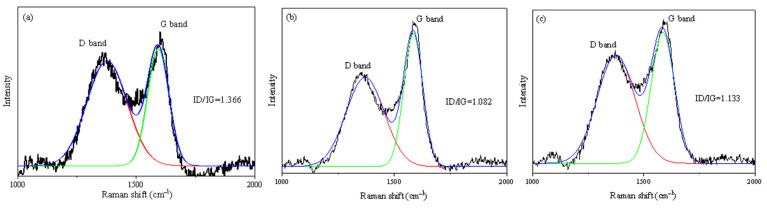
Raman spectrum of the char residues for (**a**) pure EP, (**b**) EP/7%PPAP, and (**c**) EP/6%PPAP/1%CNNS.

Moreover, the degree of graphitization was evaluated by Raman spectroscopy, and the results were shown in Figure 8. It was previously accepted that the G band represents the in-plane stretching vibration of the sp^2^ hybridization of the C atom, while the D band represents the defect in the C atom lattice. The amount of graphitization of carbon materials was frequently determined by the intensity area ratio of the D band and G band. A lower ID/IG number denotes a better degree of graphitization and fewer defect levels [38]. The thermal stability and compactness of the carbon residue with a high degree of graphitization were both very high, effectively preventing the exchange of heat, oxygen, and fuel, and enhancing the carbon residue’s flame retardancy. As shown in Figure 8, near 1360 and 1590 cm^−1^, there were two obvious Raman peaks in the spectrum, namely the D band and G band. The ID/IG of pure EP was as high as 1.366, while the ID/IG of EP/7%PPAP and EP/6%PPAP/1%CNNS were reduced to 1.082 and 1.133, respectively. On the one hand, PPAP has an excellent ability to catalyze carbon formation. On the other hand, CNNS provided a rich carbon source and a physical barrier effect; therefore, EP/7%PPAP and EP/6%PPAP/1%CNNS had the same efficiency flame retardant performance in an intumescent flame retardant system.

### 3.4. DMA Analysis of EP Composites

**Figure 9:**
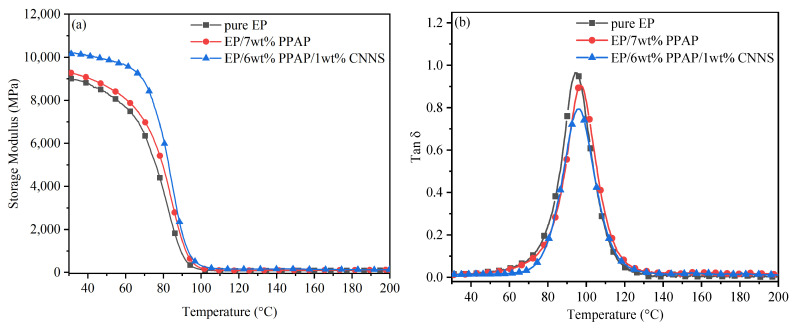
Storage modulus (**a**) and tan delta (**b**) curves for EP composites.

Figure 9 displays the temperature dependence of the storage modulus and mechanical loss factor tan δ of pure epoxy resin and its composites. The introduction of PPAP slightly increased EP’s energy storage modulus from 9004.9 MPa to 9276.4 MPa. When PPAP/CNNS were added into EP, the energy storage modulus of the material significantly increased to 10,160 MPa, which was because the uniformly dispersed CNNS enhanced the crosslinking density and increased the stiffness of the polymer. It restricted the migration of the molecular chain and gave the composites a high mechanical deformation resistance. When the temperature exceeded the glass transition temperature, the matrix became a viscoelastic material. Therefore, the matrix molecules had a higher mobility and lower mechanical load resistance on the filler. As can be seen from Figure 9b, the addition of PPAP and CNNS slightly increased the glass transition temperature of EP. The glass transition temperatures of EP/7 wt% PPAP and EP/6 wt%PPAP/1 wt%CNNS were increased from 95 °C to 96.8 °C and 97.6 °C, respectively. The tan δ peak value was the lowest at EP/6 wt%PPAP/1 wt%CNNS and the highest at pure epoxy resin. The peak value of tan δ represented the energy dissipation under cyclic loading and was related to the interaction between fillers and polymer molecules. The minimum value of tan δ at EP/6 wt%PPAP /1 wt%CNNS indicated that the material can respond to the stress in time when the glass transition temperature was reached.

## 4. Discussion

**Scheme 2:**
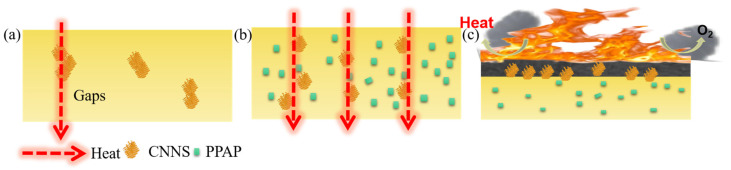
Schematic illustration of the thermal conductivity of (**a**) EP/CNNS and (**b**) EP/PPAP/CNNS, and (**c**) the flame retardation mechanisms of EP/PPAP/CNNS.

The thermal conductivity and flame retardant mechanism of EP/PPAP/CNNS are discussed next. In prior findings, synergistic enhancing effects in TC by several fillers were typically described as improving dispersion and interfacial contact, having a bridging effect, and so on [25,33]. According to the above thermal conductivity analyses of EP composites, the suggested thermal conductive mechanisms of CNNS and PPAP/CNNS on the EP matrix were illustrated in Scheme 2a,b. As shown in Scheme 2a, the stacking and aggregation behaviors of CNNS sheets in the matrix meant that it was hard to form continuous thermal conductive paths, which still induce the low TC of EP/CNNS. The behavior could be effectively suppressed by further adding the PPAP/CNNS hybrid into EP due to the good dispersion performance of fillers in the matrix slurry, as described above (Scheme 2b). It was feasible to create a continuous effective heat conductance network in EP/PAP/CNNS in combination with the CNNS bridging connection, which results in a significant synergistic improvement in the TC of the composite at the same CNNS loading. The synergistic flame retardant mechanism between CNNS sheets and the PPAP hybrid in the EP matrix is shown in Scheme 2 in accordance with the prior analyses. Firstly, while burning EP/PPAP/CNNS, the flame retardant properties of PPAP and the barrier properties of CNNS may lower the burning temperature, decrease the concentration of combustible gases and oxygen, and boost the production of organic char. Secondly, CNNS sheets produce a “tortuous path” effect to stop the transfer of heat, oxygen, and volatile chemicals when combined with the developing organic chars [35]. As a result, the EP/PPAP/CNNS composite showed a noticeable improvement in flame retardancy because of the compact and durable protective char layer created by catalytic carbonization of PPAP and the template effect of CNNS.

## 5. Conclusions

In summary, this work presented thermal oxygen etching to prepare the CNNS. As expected, the CNNS and PPAP were incorporated into the EP matrix to prepare composites through the melt blending method. As expected, the incorporation of the CNNS and PPAP into EP composites with a filling amount of 1 wt% resulted in better thermal conductivity by improving the dispersion and interfacial interactions. When it was compounded with PPAP, it had a better flame retardant performance, and its flame retardant coefficients HRR, THR, SPR, and TSP were greatly improved to 63.7%, 54.2%, 17.9%, and 57.2% reductions, respectively, compared with pure EP. These improvements in flame retardation can be attributed to the gas-phase mechanism, in which phosphorus-containing compounds from PPAP form large amounts of radical-capture agents upon exposure to the flame, and the physical barrier effects of CNNS. It offers a potentially effective method for creating thermally conductive polymer-based materials that are highly flame-resistant.

## Figures and Tables

**Table 1:** Thermal degradation date of epoxy resin composites.

Sample	Tinitial (°C)	Rpeak1/Tpeak1 (%/min/°C)	Rpeak2/Tpeak2 (%/min/°C)	Char Residue (wt%) 800 °C
Pure EP	327.9	12.1/367.9	1.4/644.6	0.3
EP/7 wt% PPAP	315.7	14.9/347.0	-	17.9
EP/6 wt%PPAP/1 wt%CNNS	317.9	15.4/349.7	-	16.7

**Table 2:** Composition, thermal conductivity, and flame retardant properties of epoxy resin.

E-51 (g)	CNNS (wt %)	PPAP (wt %)	Thermal Conductivity (W·m^−1^k^−1^)	UL-94	LOI (%)
100	-	5	-	NR	27.2
100	-	6	-	V-1	28.4
100	-	7	-	V-0	29.7
100	0.5	6.5	0.844	V-0	29.5
100	1	6	1.118	V-0	28.8
100	1.5	5.5	1.164	V-1	27.6

**Table 3:** CONE data for EP and its composites.

Properties	Pure EP	EP/7%PPAP	EP/1%CNNS/6%PPAP
TTI (s)	43	29	36
PHRR (kW·m^−2^)	2123	723	771
tp (s)	95	81	108
THR (MJ·m^−2^)	124.1	58.7	56.8
PSPR (m^2^·s^−1^)	0.28	0.23	0.22
tp (s)	76	37	89
TSP (m^2^)	23.5	10.1	10.3
Av-EHC (MJ·kg^−1^)	25.2	7.5	7.8
Char residue (%)	4.1	39.3	37.8

## Data Availability

Not applicable.
